# De novo assembly and transcriptome dataset of liver, testis and head kidney from red drum (*Sciaenops ocellatus*)

**DOI:** 10.1016/j.dib.2019.01.011

**Published:** 2019-01-11

**Authors:** Tracy A. Sherwood, Kevan L. Main, Dana L. Wetzel

**Affiliations:** Mote Marine Laboratory, 1600 Ken Thompson Pkwy, Sarasota, FL 34235, USA

## Abstract

Red drum (*Sciaenops ocellatus*) is an estuarine Sciaenid with high commercial value and recreational demand. During the past 50 years, overfishing has caused declines in the population that resulted in the development of red drum commercial and stock enhancement aquaculture fisheries. Despite the potential high economic value in both wild and aquaculture commercial fisheries the availability of transcriptomic data for red drum in public databases is limited. The data here represents the transcriptome profiles of three tissues: liver, testis and head kidney from red drum. The data was generated using Illumina high-throughput RNA sequencing, Trinity for de novo assembly and Blast2GO for annotation. Six individual libraries were pooled for sequencing of the transcriptome and the raw fastq reads have been deposited in the NCBI-SRA database (accession number SRP11690).

**Specifications table**

**Table:** 

Subject area	*Biology*
More specific subject area	*Marine fisheries, Aquaculture*
Type of data	*Table, figure,*Supplementary tables
How data was acquired	*Illumina HiSeq. 2500*
Data format	*Raw (FastQ)*
Experimental factors	*Liver, testis and head kidney collected from male red drum*
Experimental features	*De novo assembly and transcriptome analysis*
Data source location	*Mote Aquaculture Research Park, Sarasota, FL, 34240, USA*
Data accessibility	*Data is available in the article and at*https://www.ncbi.nlm.nih.gov/sra/SRP116909
Related research article	*Xu, E.G. et al., (2017)’Larval red drum (Sciaenops ocellatus) sublethal exposure to weathered deepwater horizon crude oil: development and transcriptomic consequences’ Environmental Science and Technology 51(17) 10162–10172*doi:10.1021/acs.est.7b02037

**Value of the data**

• This data adds to the existing red drum transcriptomic data available in the public database NCBI-SRA (Accession no. PRJNA357008, PRJNA488237).
• Identifying and annotating red drum transcriptomes from different organs provides basic functional genomic information for each organ.
• The data will enhance opportunities for genetic improvement of red drum for aquaculture production and stock enhancements of wild populations.

## 1 Data

**Fig. 1:**
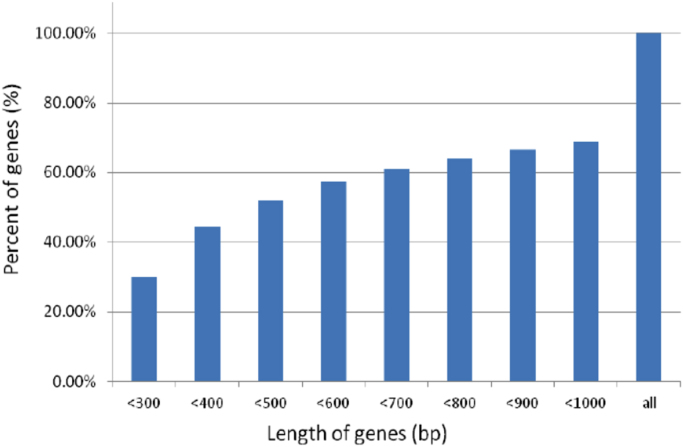
Size distribution of 116,036 *de novo* assembled genes.

**Fig. 2:**
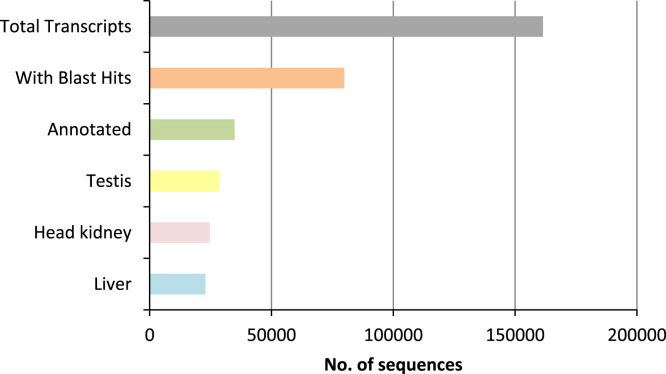
Total number of *de novo* assembled transcripts with blast hits (79,927), GO annotations (34,841) and annotations for each tissue: testis (28,482), head kidney (24,663) and liver (22,922).

**Fig. 3:**
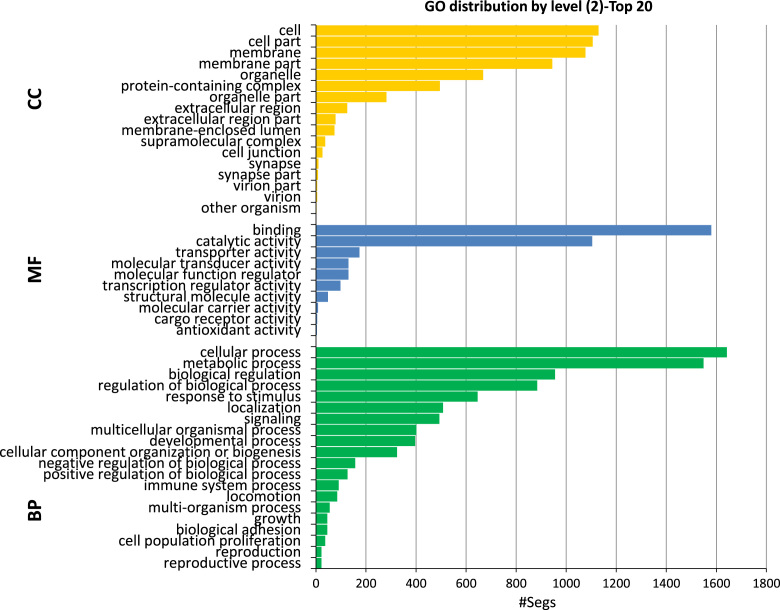
GO distribution by level (2) of the red drum transcripts based on three main levels; biological processes (BP), molecular function (MF), and cellular component (CC).

**Fig. 4:**
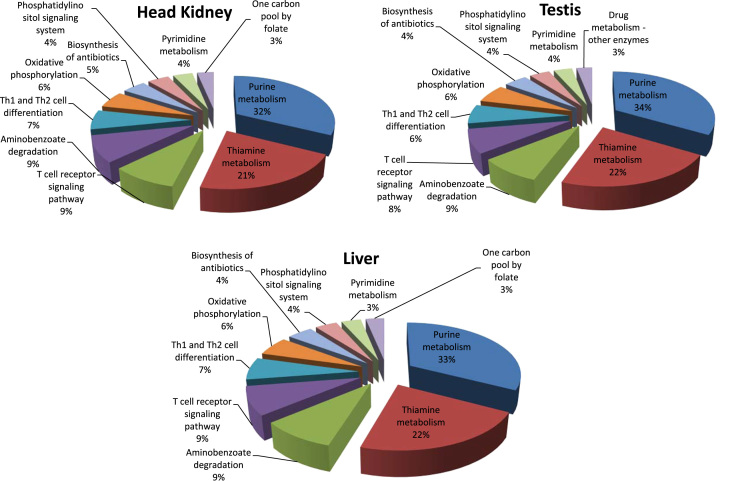
KEGG pathway analysis of the top 10 pathways showing the percentage of enzyme sequences present in the three tissues; liver, testis and head kidney. Percentages represent the number of sequences assigned within the biological pathways.

**Fig. 5:**
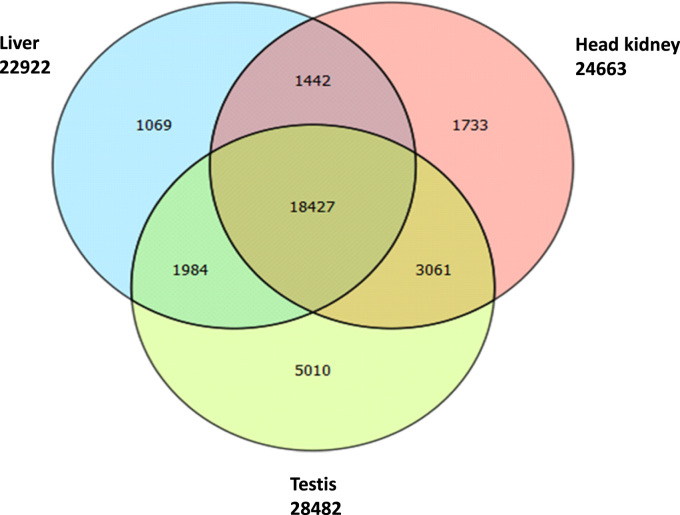
Venn diagram representing the number *de novo* annotated transcripts that are common and uncommon among the tissues; liver, testis and head kidney.

High throughput deep RNA-sequencing was done to generate a *de novo* reference sequence for red drum using cDNA libraries constructed from liver, testis and head kidney tissues. Sequencing on the HiSeq. 2500 generated a total of 128.9 million paired-end reads with an average length of 100 bp. Trinity *de novo* assembly produced 161,438 transcripts and 116,036 genes with a Contig N50 of 2224 bp (Table 1). Size distribution of the 116,036 genes showed that 70% of the *de novo* assembled genes were greater than 1000 bp (Fig. 1). Gene ontology (GO) analysis of the 161,438 transcripts was done using the Blast2GO® blastx program against the non-redundant vertebrata database of NCBI with the default cutoff e-value of 10^−3^. This analysis generated 79,927 (49.5%) transcripts with blast hits to known proteins, of those 34,841 (21.5%) transcripts were assigned at least one functional GO term (Fig. 2 and S1). The annotated transcripts were categorized by GO distribution level (2) with the top 20 categories of one of three main levels: biological process, molecular function, and cellular components (Fig. 3). KEGG pathway analysis was done to understand the biological processes of the annotated transcripts for each tissue. The number of transcripts assigned enzyme codes to run the KEGG pathway analysis were 769 for liver, 817 for head kidney, and 860 for gonad (Fig. 4 and S2). Tissue distribution of all identified transcripts by the three tissues is presented in (Fig. 5 and S3), showing the majority (18,427, 56%) were expressed in all the tissues.

**Table 1:** *De novo* transcriptome assembly.

Total raw reads (all tissues)	129,918,744
Total clean raw read (all tissues)	128,979,868
Total trinity ‘genes	116,036
Total trinity transcripts	161,438
Percent GC	47.09
Contig N50^a^	2224
Median length	467
Average contig	1068
Total assembled bases	172,435,728

a The contig N50 value is defined as the maximum length whereby at least 50% of the assembled sequence resides in contigs of at least that length. Raw reads were trimmed based on base quality. Sliding windows along with quality and length thresholds were used to trim the 3׳- and 5׳-end of reads. Sequences with Ns were also removed. Data Phred score was >25.

## 2 Experimental design, materials, and methods

### 2.1 Fish, tissue sampling and RNA extraction

Red drum were reared from captive spawned broodstock and obtained from Mote Aquaculture Research Park (Sarasota, FL). Liver, testis and head kidney tissues were aseptically dissected from two adult red drum males (weight 755–899, length 39–42 cm), euthanized using MS-222 at 300 ppm for 15 min. At sacrifice, tissues were rinsed with PBS and placed immediately in RNA*later*™ (Sigma-Aldrich Corp., USA). Total RNA was extracted from 30 mg of tissue using Tri-Reagent® (Sigma-Aldrich Corp., USA) following manufacturer׳s instructions. RNA quantity was evaluated by the Qubit 3.0 Fluorometer (Life Technologies, USA) and quality with the 2100 Bioanalyzer (Agilent, USA). Only samples with an RIN >7.0 were used for sequencing. One μg of total RNA from each tissue sample was sent for RNA sequencing (Omega Biotek Inc., Norcross, GA).

### 2.2 RNA sequencing, de novo assembly, and transcript annotation

Construction of the RNA-seq libraries, Illumina RNA sequencing and *de novo* assembly were carried out by Omega Bioservices (Omega Biotek Inc., Norcross, GA). Briefly, paired-end 100 bp sequencing was performed on the Illumina HiSeq. 2500 sequencer. The sequencing quality was assessed using FASTQC (0.10.1). Data was filtered using Trimmomatic v0.30, removal of primer and adaptor sequence, truncation of sequence reads with both pair end quality < 25, truncation of sequence reads not having an average quality of 25 over a 4 bp sliding window based on the phred algorithm. All reads were combined to assemble a *de novo* transcriptome for red drum using Trinity [1]. The trimmed reads from each sample were aligned to the assembled transcriptome, separately. The expression abundance for genes/transcripts was calculated using eXpress [2]. Expression levels of genes/transcripts were normalized across samples using the trimmed mean of M-values normalization method (TMM). Gene ontology and Kyoto Encyclopedia of Genes and Genomes (KEGG) pathway analysis were determined using Blast2GO® PRO version 4.1.7 [3]. Tissue distribution of the transcripts was evaluated using the open access Orange version 3.4.5 [4].

## References

[1] Grabherr M.G., Brian J. Haas, Levin Moran Yassour Joshua Z., Thompson Dawn A., Amit Ido, Adiconis Xian, Fan Lin, Raychowdhury Raktima, Zeng Qiandong, Chen Zehua, Mauceli Evan, Hacohen Nir, Gnirke Andreas, Rhind Nicholas, di Palma Federica, Friedman Bruce W.N., Trinity A.R. (2013). Reconstructing a full-length transcriptome without a genome from RNA-Seq data. Nat. Biotechnol..

[2] Roberts A., Pachter L. (2013). Streaming fragment assignment for real-time analysis of sequencing experiments. Nat. Methods..

[3] Conesa A., Götz S., García-Gómez J.M., Terol J., Talón M., Robles M. (2005). Blast2GO: a universal tool for annotation, visualization and analysis in functional genomics research. Bioinformatics..

[4] Demšar J., Curk T., Erjavec A. (2013). Orange: data mining toolbox in Python. J Mach. Learn Res.

